# Hydroxychloroquine and chloroquine retinal safety concerns during COVID-19 outbreak

**DOI:** 10.1007/s10792-020-01593-0

**Published:** 2020-09-18

**Authors:** Massimo Nicolò, Lorenzo Ferro Desideri, Matteo Bassetti, Carlo Enrico Traverso

**Affiliations:** 1 IRCCS Ospedale Policlinico San Martino, University Eye Clinic of Genoa, Genova, Italy; 2 Department of Neurosciences, Rehabilitation, Ophthalmology, Genetics, Maternal and Child Health (DiNOGMI), University of Genoa, Genova, Italy; 3 Macula Onlus Foundation, Genova, Italy; 4 Infectious Diseases Clinic, Department of Health Sciences, University of Genova and Ospedale Policlinico San Martino- IRCCS Per L’Oncologia, Genova, Italy

**Keywords:** Hydroxychloroquine, Chloroquine, Retina, Retinal safety, COVID-19, Coronavirus, SARCoV-2

## Abstract

**Purpose:**

The current coronavirus disease 2019 (COVID-19) has been declared by the World Health Organization a global pandemic. Chloroquine (CQ) and hydroxychloroquine (HCQ) have been largely adopted in the clinical setting for the management of SARS-CoV-2 infection; however, their known retinal toxicity has raised some safety concerns, especially considering the higher-dosage employed for COVID-19 patients as compared with their suggested posology for their usual indications, including systemic lupus erythematosus and other rheumatic diseases.

In this review, we will discuss the optimal dosages recommended for COVID-19 patients when treated with HCQ and CQ.

**Methods:**

A comprehensive literature search was performed in PubMed, Cochrane library, Embase and Scopus, by using the following search terms: "chloroquine retinal toxicity" and "hydroxychloroquine retinal toxicity" alone or in combination with "coronavirus", "COVID-19", " SARS-CoV-2 infection " from inception to August 2020.

**Results:**

Although there is still no consistent evidence about HCQ/CQ retinal toxicity in patients with COVID-19, these possible drug-related retinal adverse events may represent a major safety concern. For this reason, appropriate screening strategies, including telemedicine, should be developed in the near future.

**Conclusion:**

A possible future clinical perspective for patients with COVID-19 treated with HCQ/CQ could reside in the multidisciplinary collaboration between ophthalmologists monitoring the risk of HCQ/CQ-related retinal toxicity and those physicians treating COVID-19 infection.

## Introduction

The current coronavirus disease 2019 (COVID-19) has been declared by the World Health Organization (WHO) a global pandemic in early March 2020 [1]. To date, no specific therapy has been identified for treating COVID-19; however, beside antiviral drugs, chloroquine (CQ) and hydroxychloroquine (HCQ) have been employed for the management of SARS-CoV-2 infection [2]. In this regard, HCQ and CQ belong to the class of synthetic antimalarial drugs, which are generated from the bark of cinchona (Rubiaceae) are characterized by a water solubility (HCQ is more soluble given its hydroxyl group) and a long plasma elimination half-life (t_1/2_) of 1300 and 900 h, respectively [3]. Their antiviral properties are thought to reside in their capacity to increase the lysosomes, endosomes and Golgi complex pH due to the accumulation of aminoquinolines and therefore by making viral replication more difficult [4].

I*n vitro* studies showed HCQ and CQ efficacy in inhibiting SARS-CoV-2 replication.

[5] [6]. To date, there are more than 20 ongoing randomized clinical trials (RCTs) on both medications for treatment of COVID-19 [7]. In particular, Gao et al. demonstrated the clinical efficacy of CQ in reducing pneumonia exacerbation and improving lung imaging findings in more than 100 patients [8]. Similarly, Gautret et al. showed that HCQ was effective in reducing viral load in 20 patients with COVID-19 [9].

Given this background, the Italian Society of Infectious and Tropical disease recommended the administration of CQ 500 mg × 2/die or HCQ 200 mg/die for 10 days, with possible extension to 20 days according to the severity of the disease [10]; similarly, the Dutch Center of Disease control (CDC) suggested in adults a regimen with 600 mg of CQ followed by 300 mg after 12 h on day 1, then 300 mg × 2/die per os on days 2–5 days [11]. In this review, we will analyze all the studies dealing with HCQ and CQ-related retinal toxicity, especially focusing on the setting of patients with COVID-19 infection. In particular, we will discuss the possible role of screening strategies for the early detection of HCQ/CQ-related retinal toxicity in these patients and the importance of a multidisciplinary collaboration between ophthalmologists and those doctors treating COVD-19 infection.

## Methods

A literature search was performed in order to find all the published studies dealing with HCQ and CQ retinal toxicity from inception until August 2020. The following electronic databases were adopted: Medline, PubMed, Science Citation Index via Web of Science, and the Cochrane Library. The following search terms were used: ‘chloroquine retinal toxicity" and "hydroxychloroquine retinal toxicity" alone or in combination with "coronavirus", "covid 19", " SARS-CoV-2 infection ". Moreover, all the articles were thoroughly evaluated and their reference lists were also studied in order to find other manuscripts that could be included in this present review study.

### HCQ and CQ retinal toxicity

It has been largely reported that CQ and HCQ may display a retinal toxic effect when used for a long time in patients lupus erythematosus (SLE) and other rheumatoid diseases.

The lower toxicity of HCQ compared with CQ has been referred to the presence of the hydroxyl group limiting the ability of HCQ to cross the blood–retinal barrier [12]. Preclinical studies showed that HCQ and CQ are melanotropic drugs, binding to the melanin of the retinal pigmented epithelium (EPR); however, it has not fully understood the pathogenetic mechanism, which is thought to primarily occur in the neural retina (ganglion cells and photoreceptors), with subsequent alterations in the RPE showing intracellular accumulation of lipofuscin [13]. In a recent in vitro study, HCQ and CQ were shown to block the uptake activity of an organic anion transporting polypeptide 1A2 (OATP1A2), which is expressed in human RPE cells and is involved in the recycling of all-trans-retinol. In fact, HCQ inhibited significantly the uptake of all-trans-retinol in human embryonic kidney cells (HEK293) and primary human RPE cells. This study would suggest the effect of HCQ and CQ on the visual cycle function, whose alteration may lead to vision impairment and to development of the retinopathy clinically observed [14]. From a clinical perspective, CQ and HCQ retinal toxicity is usually asymptomatic with the preservation of visual acuity in the early stages. In more advanced stages, patients may experience a decreased visual acuity and peripheral vision, alterations in night vision until the formation of a paracentral scotoma [15]. A pathognomonic clinical sign, characterized by the onset of a “bull's eye”, is due the formation of a perifoveal ring of retinal pigment epithelium (RPE) atrophy sparing the fovea and it usually causes irreversible visual loss [12].

Interestingly, some ethnic differences have been reported, since HCQ retinopathy typically presents with a pericentral pattern (instead of the parafoveal bull’s eye) in Asian patients [16].

A retrospective case–control study on 2361 patients under protracted HCQ treatment for at least 5 years reported an overall prevalence of HCQ-related retinopathy of 7.5%, varying in relation to the daily dosage. In particular, with a dosage ranging from 4.0 to 5.0 mg/kg, the prevalence of retinal toxicity was less than 2% within the first 10 years of treatment but increased to almost 20% after 20 years [17]. By contrast, a larger scale study including data from the National Data Bank for Rheumatic Diseases on 3995 patients with SLE or rheumatoid arthritis (RA) treated with HCQ and found 298 patients reporting a diagnosis of HCQ-induced retinopathy. After multiple imputation for missing data, the authors revealed an overall risk of retinopathy of 0.65%, in particular 1.0% after 10 years of treatment and 3.1% at 20 years of use. The lower prevalence of HCQ-induced retinopathy reported in this study was probably due to the presence of missing data and the adoption of older screening modalities [18].

Moreover, other known risk factors associated with HCQ-related retinopathy are the presence of a concomitant renal disease, use of tamoxifen and other macular diseases [15].

Considering that this iatrogenic retinopathy is referred to be associated with a high dosage and long-term treatment period, the American Academy of Ophthalmology (AAO) has recommended a maximum daily dose ≤ 5.0 mg/kg real body weight for HCQ and ≤ 2.3 mg/kg real body weight for CQ [15].

Similarly, the British Royal College of Ophthalmologists (RCO) has indicated a daily dose of HCR inferior to 5 mg/kg/day for less than 5 years as relatively safe for retinal toxicity [19].

According to the AAO, a retinal screening is recommended at baseline and within the first year and five years for the treatment with CQ and HCQ, respectively [15]; moreover, both AAO and RCO underlined that the retinal screening should be performed earlier if the major risk factors are present [15] [19].

The current doses proposed for treating COVID-19 are 4–5 times higher than those suggested by AAO and RCO [20]. In this regard, studies investigating the role of HCQ in non-rheumatoid diseases revealed an incidence of drug-related retinopathy ranging from 25 to 40% within the year of treatment [15]. In particular, Leung et al*.* reported the rapid incidence of HCQ-related retinopathy in 2 out of 7 oncologic patients treated with HCQ 1000 mg for lung cancer. Signs of retinopathy were demonstrated by spectral-domain optical coherence tomography (OCT) and multifocal electroretinography (mfERG). Although a possible synergic retinal toxic effect of HCQ with erlotinib could have not been ruled out, this study proved that high doses of HCQ may lead to retinal toxicity within 1–2 years of treatment [21].

In another cohort study, retinal toxicity was examined in 12 patients with chronic graft-versus-host disease treated with high doses of HCQ (median 11.5 mg/kg/day). 25% of the patients (n = 3/12) showed retinal toxicity with the onset of scotomas in the Amsler grid and Humphrey 10–2 automated perimetry. Hence, also this study showed the association between higher doses of HCQ and an increased and earlier incidence of drug-related retinopathy [22].

Moreover, it is known that chronic CQ abusers are highly susceptible to retinal damage; one study on patients with heart block revealed that CQ chronic abuse was associated with the onset of CQ-related retinopathy in 53.8% of them [23]. (Table 1).

**Table 1:** Recommended HCQ and CQ dosage released by the various international ophthalmological societies regarding preventing drug-induced retinal toxicity

	Dutch guidelines	Belgian guidelines	Italian guidelines	Chinese guidelines	Thailandese guidelines
Recommeneded HCQ dosage	800 mg at day 1, then 400 mg/day up to 5 days	800 mg at day 1, then 400 mg/day up to 5 days	800 mg at day 1, then 400 mg/day up to 10 days	N/A	800 mg at day 1, then 400 mg/day for at least 5 days
Recommeneded CQ dosage	900 mg at day 1, then 600 mg/day up to 5 days	900 mg at day 1, then 600 mg/day up to 5 days	1000 mg/day up to 10 days	1000 mg/day up to 10 days	500/mg day for at least 5 days

CQ–Chloroquine; HCQ–Hydroxychloroquine

### Screening and diagnostic modalities

In the last years, several both structural and functional, diagnostic techniques have been investigated for the screening of HCQ-related retinopathy [24].

Among them, the AAO guidelines (2016) recommended the combination between a functional test, the automated 10–2 VFA and a structural imaging technique, the spectral domain optical coherence tomography (SD-OCT), as a first choice to detect early signs of HCQ-induced retinopathy [25].

The earliest signs of retinopathy detected on the central visual field by 10–2 VFA are represented by a cluster of paracentral points with decreased sensitivity. With the progress of the disease, a partial bull’s eye scotoma resembling an arcuate defect may appear and subsequently developing into a complete bull's eye scotoma with a full ring defect sparing the fovea [26].

On the other hand, SD-OCT imaging can detect early alterations occurring in the retinal layers before the onset of a clinically visible HCQ- related retinopathy; in particular, early alterations are visualized as a disruption of the outer nuclear layer, external limiting membrane and RPE in the parafoveal area [27]. A distinctive sign of HCQ-induced retinopathy is the “flying saucer sign”, which is caused by the preservation of the central subfoveal architecture at the detriment of the atrophy of the contiguous perifoveal outer retinal tissue [28].

While SD-OCT has been proven to be objective and highly specific, by contrast, the 10–2 VFA examination is a subjective tool and it has been revealed to be more sensitive than SD-OCT in detecting the earliest HCQ-induced alterations occurring in retinal layers [25].

In this regard, a retrospective study on 121 patients taking HCQ or CQ showed that SD-OCT alone had a sensitivity of 78.6% and a specificity of 98.1%, while for 10–2 VFA were 85.7% and 92.5%, respectively. Moreover, the combination between 10 and 2 VFA and SD-OCT examinations improved the overall sensitivity and specificity to 85.7% and 92.5%, respectively [29].

In addition, multifocal electroretinography (mfERG) has been revealed to be a very sensitive screening test to detect early alterations in HCQ-related retinopathy, in particular by showing the paracentral reductions in amplitude, which are considered to be the most specific waveform pattern of this iatrogenic retinopathy; however, considering the cost, the limited access, the need of a well-trained technician staff and the importance of patient’s collaboration, mfERG should not be considered as a first choice screening modality [30].

Fundus autofluorescence (FAF) is an in vivo imaging technique, detecting the distribution of lipofuscin in the outer retina, subretinal space and RPE. In patients treated with HCQ, FAF may detect an augmented signal intensity due to the altered distribution secondary to the early damage in the photoreceptor layer. Similarly to mfERG, given its operator-dependent nature, FAF should be considered more a complementary screening test rather than a primary choice [31].

Fundus photography can be considered a helpful descriptive tool in advanced stages of the disease, because visible HCQ-related retinopathy is often a late clinical finding. Thus, the AAO did not recommend fundus photography as a first choice for screening HCQ-induced retinopathy [26].

Lastly, other diagnostic modalities such as color vision assessments, Amsler grid, fluorescein angiography (FA) and electro-oculography are not currently recommended as primary screening examinations, since they have not been proven to be sufficiently sensitive for detecting HCQ-related retinopathy [32].

### Clinical perspectives during covid-19 outbreak

To date, it has not been demonstrated if the HCQ or CQ exposure at higher doses over a short treatment period is likewise associated with comparable retinal toxicity as with the chronic exposure. In addition, other main concerns raised by the EU Eye for the management of COVID-19 patients with HCQ and CQ are the presence of a polypharmacy and the combination between high doses and a high comorbidity, respectively [33].

The main issues are represented by the risk of overdosing HCQ and CQ in COVID-19 because of the relatively low awareness among doctors about this iatrogenic toxicity, in combination with the lack of evidence about a fixed regimen of these drugs adopted in COVID-19 patients, and, nonetheless, the faster deterioration in COVID-19 patients under mechanical ventilation, which has been reported to reduce muscle mass by 2.4% per day, leading to the increase of skeletal muscle creatinine kinase levels [34]. In this regard, pharmacokinetic studies have demonstrated the large volume of distribution (V_d_) of HCQ, which is distributed across a variety of tissues, including fatty tissues [35]; for this reason, there is still a debate about the optimal HCQ dosing regimen in relation to the weight measurements used in these calculations, and in particular between the ideal body weight (IBW) and the adjusted body weight (ABW) [36].

Furthermore, as suggested by the Belgian guidelines for COVID-19 management, those patients with a medical history of maculopathy or other retinal diseases should be screened before HCQ/CQ treatment, in order not to neglect an important contraindication possibly causing toxic retinopathy [37].

Currently, there is still no general consensus about the optimal dosage and duration of the treatment with HCQ/CQ for COVID-19 patients, doses are generally higher than those recommended for systemic diseases and there are no homogenous guidelines on the possibility to recontrol and module the dosage during the treatment. In this regard, the current clinical trials on HCQ/CQ should carefully consider the possibility of seasonal recurrences of SARS-CoV-2 infection, which would lead to the adoption of HCQ/CQ cumulative doses and therefore a higher risk of retinal toxicity.

We recognize that a traditional on site ophthalmologic visit, usually considered mandatory, could not be realistic in hospitalized patients with COVID-19 needing a primary support and often mechanical ventilation.

However, trying to transform the COVID-19 problem in an opportunity, we should rethink HCQ or CQ retinal screening visit also to be feasible in hospitalized COVID-19 patients or after recovery. Currently, OCT reporting the ganglion cell complex (GCL), retinal nerve fiber layer (RNFL) and other retinal layers thicknesses and 10–2 VFA examinations as primary screening tests, in combination with other additional imaging modalities (FAF and fundus photography), can be easily performed by trained technicians like nurses working in COVID-19 division, who can acquire electronic retinal images to be evaluated remotely afterward by an ophthalmologist trained in retinal disease.

In this regard, in the last years the concept of telemedicine has been rapidly growing in the ophthalmological field, in order to facilitate the evaluation, diagnosis, and management of remote patients [38]. For example, the SUNDROP Study, a retrospective analysis in more than 1000 premature newborns, revealed that telemedicine had a sensitivity of 100% and specificity of 99.8% for screening the retinopathy of prematurity (ROP) [39]. Moreover, given the high percentage of diabetic patients unaware of their diabetic retinopathy (almost 70% of them), many screening programs have been investigated in terms of the cost-effectiveness and reliability of telemedicine for this disease, showing promising results in this respect [40]. In this way, fundus photographs and OCT examinations are taken by nursing staff or technicians and forwarded to a remote reading center, where the ophthalmologist can make the diagnosis and write a medical report for the follow-up and further clinical evaluation of the patient [41].

Interestingly, the role of telemedicine is being studied with encouraging results also for the screening of other common ocular diseases, including age-related macular degeneration and glaucoma [42] [43].

Hence, given this evidence and the increasing adoption in the clinical practice of HCQ for treating COVD-19, in the near future, further clinical studies should better investigate the role of telemedicine for the screening of HCQ-related retinopathy. This scenario would lead to the necessity to establish a multidisciplinary collaboration between ophthalmologists and those physicians providing the primary care for COVID-19.

## Conclusion

Although there is still no consistent evidence about the clinical efficacy of HCQ and CQ for treating COVID-19, these 2 drugs have been largely employed in the clinical setting; moreover, several, new clinical trials are investigating the role of HCQ and CQ for the treatment of COVID-19 patients. Given this background, it is likely that HCQ and CQ retinal toxicity could represent a non-negligible issue in the near future. In this regard, further larger-scale clinical studies should provide a more detailed evidence of the HCQ/CQ-related retinal toxicity in COVID-19 patients, in order to better address their clinical management from an ophthalmological perspective. Given the critical issue and possible contagiousness, COVID-19 division should be equipped with the necessary instruments need to screen retinal features for possible HCQ or CQ toxicity diagnosed remotely. The multidisciplinary collaboration between eye doctors and those physicians treating COVID-19 infection will probably represent an important issue to handle in the near future.
